# Acute Respiratory Distress Syndrome and the Use of Inhaled Pulmonary Vasodilators in the COVID-19 Era: A Narrative Review

**DOI:** 10.3390/life12111766

**Published:** 2022-11-02

**Authors:** Adeel Nasrullah, Shiza Virk, Aaisha Shah, Max Jacobs, Amina Hamza, Abu Baker Sheikh, Anam Javed, Muhammad Ali Butt, Swathi Sangli

**Affiliations:** 1 Division of Pulmonology and Critical Care, Allegheny Health Network, Pittsburgh, PA 15212, USA; 2 Department of Internal Medicine, Allegheny Health Network, Pittsburgh, PA 15512, USA; 3 Department of Internal Medicine, University of New Mexico, Albuquerque, NM 87106, USA

**Keywords:** acute respiratory distress syndrome, ARDS, COVID-19, COVID acute respiratory distress syndrome, CARDS

## Abstract

The Coronavirus disease (COVID-19) pandemic of 2019 has resulted in significant morbidity and mortality, especially from severe acute respiratory distress syndrome (ARDS). As of September 2022, more than 6.5 million patients have died globally, and up to 5% required intensive care unit treatment. COVID-19-associated ARDS (CARDS) differs from the typical ARDS due to distinct pathology involving the pulmonary vasculature endothelium, resulting in diffuse thrombi in the pulmonary circulation and impaired gas exchange. The National Institute of Health and the Society of Critical Care Medicine recommend lung-protective ventilation, prone ventilation, and neuromuscular blockade as needed. Further, a trial of pulmonary vasodilators is suggested for those who develop refractory hypoxemia. A review of the prior literature on inhaled pulmonary vasodilators in ARDS suggests only a transient improvement in oxygenation, with no mortality benefit. This narrative review aims to highlight the fundamental principles in ARDS management, delineate the fundamental differences between CARDS and ARDS, and describe the comprehensive use of inhaled pulmonary vasodilators. In addition, with the differing pathophysiology of CARDS from the typical ARDS, we sought to evaluate the current evidence regarding the use of inhaled pulmonary vasodilators in CARDS.

## 1. Introduction

The Coronavirus disease (COVID-19) pandemic of 2019 has resulted in significant morbidity and mortality, especially from severe acute respiratory distress syndrome (ARDS). COVID-19-associated ARDS (CARDS) differs from the typical ARDS due to distinct pathology involving the pulmonary vasculature endothelium, resulting in diffuse thrombi in the pulmonary circulation and impaired gas exchange. The National Institute of Health and the Society of Critical Care Medicine recommends lung-protective ventilation, prone ventilation, and neuromuscular blockade as needed. Further, a trial of pulmonary vasodilators is suggested for those who develop refractory hypoxemia. A review of the prior literature on inhaled pulmonary vasodilators in ARDS suggests only a transient improvement in oxygenation, with no mortality benefit. This narrative review aims to highlight the fundamental principles in ARDS management, delineate the fundamental differences between CARDS and ARDS, and describe the comprehensive use of inhaled pulmonary vasodilators.

## 2. Methods

The following is a review article that was formatted from a review of the existing literature. The literature search was conducted by keyword searches of the MEDLINE database accessed through PubMed. Additionally, articles were found by searching Google Scholar. As this was not a systematic review, there was not strict exclusion and inclusion criteria, with the exception of excluding articles without an available English translation. All text, tables, and images were created through Microsoft Office.

### 2.1. Definition of Acute Respiratory Distress Syndrome

**Figure 1:**
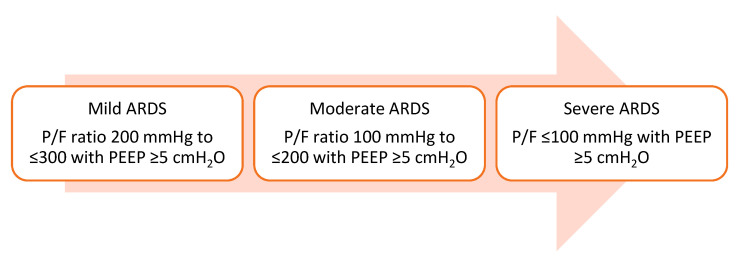
Illustrates the severity of ARDS based on the Berlin definition. P/F: PaO_2_/FiO_2_ and PEEP: positive end-expiratory pressure.

Per Berlin’s definition, acute respiratory distress syndrome (ARDS) is described as new or worsening respiratory distress within one week of a known clinical insult that presents as bilateral opacities on chest imaging that are unexplained by heart failure. It is further characterized into different severities based on abnormalities in the gas exchange based on the PaO_2_/FiO_2_ (P/F) ratio, as shown in Figure 1 [1].

### 2.2. Pathophysiology of ARDS and COVID-19-Related ARDS (CARDS)

#### 2.2.1. An Overview of the Pathogenesis of ARDS

In ARDS, vascular permeability increases from multiple mechanisms, including direct tissue injury, dysregulated molecular function, and a proinflammatory state mediated by cytokine release. As pneumocytes and pulmonary epithelium encounter direct tissue injury (i.e., infection, irritant, and trauma) or indirect endothelial damage (i.e., sepsis), resident macrophages release cytokines, leading to subsequent endothelial activation. Consequent upregulation of the chemokine pathways and release of inflammatory mediators such as reactive oxygen species, proteases, and cytokines further propagate the endothelial injury. Ultimately, this vicious cycle causes capillary leakage, generating interstitial or intra-alveolar edema build-up, which damages both pneumocytes and alveolar cells and results in compromised alveolar gas exchange and hyaline membrane formation [2]. ARDS can be characterized into various stages, as described below.

#### 2.2.2. Tissue Injury and Exudative Phase

Cell injury products or direct microbial attacks activate alveolar macrophages and trigger a cascade of immune-modulated cytokines (such as TNF) to be released and recruit neutrophils. The byproduct of such an immunomodulatory reaction is an injury to the endothelial–epithelial barrier, which leads to the loss of the typically low permeability of the alveolar membrane [3]. More specifically, neutrophils release inflammatory markers that can affect type I pneumocytes (that reabsorb alveolar fluid via active clearance) and type II pneumocytes (that secrete the surfactant to allow for gas exchange). As this occurs, alveolar spaces are filled with inflammatory-rich edema, resulting in lung de-recruitment [4].

#### 2.2.3. Proliferative Phase

As the inflammatory process evolves, the immune system reorients to repair normal alveolar function with the clearance of pathogens and damaged host cells. Key components of the proliferative phase include neutrophils apoptosis and fibroblastic expansion. If this phase is impaired, fibroblasts can continue to proliferate, resulting in irreversible fibrosis with a subsequent failure of functional alveolar recovery [5].

### 2.3. Pathogenesis of COVID-19-Associated Acute Respiratory Distress Syndrome (CARDS)

Similar to other causes of ARDS, SARS-CoV-2 causes direct viral damage and upregulates the host inflammatory and thrombotic responses both in the pulmonary and extrapulmonary systems. SARS-CoV-2 binds the angiotensin-converting enzyme 2 (ACE-2) receptor to lung epithelial cells, which internalizes the virus, infecting epithelial cells. Infected epithelial and dendritic cells with or without macrophages can activate a typical ARDS exudative response, causing diffuse damage to the alveoli, increased alveolar edema, and, ultimately, hyaline membrane formation. If unresolved, it can lead to fibrosis [6]. The mechanism of onset is typically more extended when associated with COVID-19. Unlike other causes of ARDS, CARDS is associated with macro- and micro-thrombosis, which is likely secondary to endothelial injury and is potentially facilitated by the expression of ACE2 at endothelial cells [5,7]. Early COVID-19 autopsy data showed that the pathophysiology of CARDS is more monocytic and lymphocytic, such as other viral cases of pneumonia, compared to the more neutrophilic ARDS [8]. Moreover, studies have shown that CARDS can be associated with right ventricle (RV) strain in up to 66% of mechanically ventilated patients, despite normal lung compliance, plateau pressures, and oxygenation [9]. The RV strain has been postulated to occur from direct myocardial injury and/or intravascular thrombosis. Table 1 delineates the key differences between CARDS and typical ARDS.

### 2.4. Standard Therapies for ARDS

**Figure 2:**
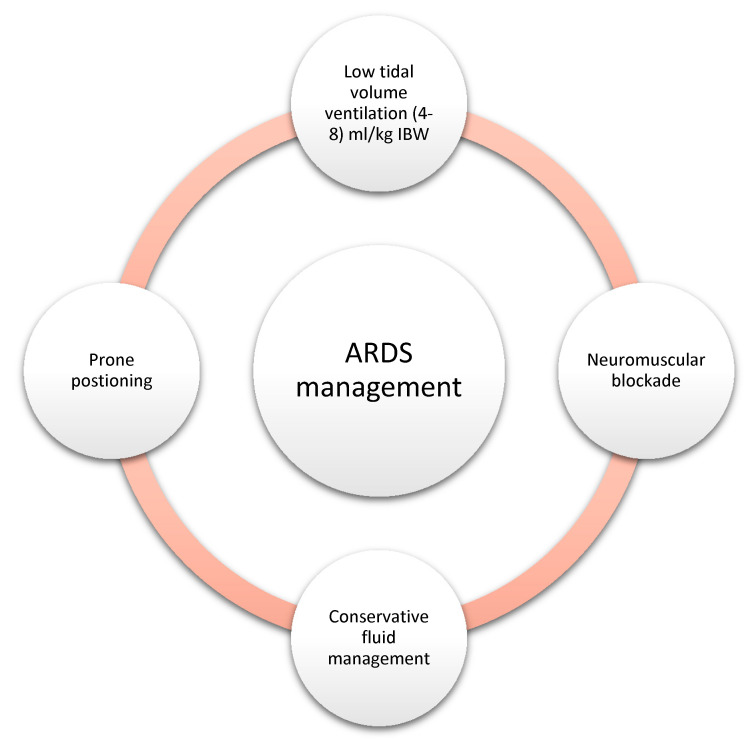
The key management strategies utilized in the management of ARDS. IBW: ideal body weight and ARDS: acute respiratory distress syndrome.

Regardless of the severity of ARDS, the standard approach to the management of ARDS includes low tidal volume ventilation [10]. Prone positioning improves ventilation-perfusion (V/Q) mismatch and has been shown to reduce the 28-day mortality in patients with moderate to severe ARDS (P/F < 150) [11]. In the same subset of patients (P/F ratio < 150), a neuromuscular blockade is also suggested, as it improves oxygenation by decreasing the ventilator desynchrony and limiting the barotrauma [12]. As sepsis is one of the most common culprits of ARDS and it does require fluid resuscitation and antibiotics administration, this can lead to volume overload. Volume overload can worsen the oxygenation by decreasing the lung compliance; hence, a conservative fluid strategy to maintain a net even fluid status has been shown to decrease the ventilatory days and intensive care unit (ICU) length of stay [13]. Moreover, in recent times, the early use of steroids in moderate to severe ARDS has suggested a reduced duration of mechanical ventilation and 28-day mortality [14]. Figure 2 summarizes the key principles of ARDS management.

Patients who remain hypoxic despite the above measures are often categorized with refractory hypoxia. There is no strict definition of refractory hypoxemia, but various studies have used a P/F ratio of less than 100–150 on PEEP > 5 cm of water with lung protective ventilation [15,16]. Different salvage therapies, such as lung recruitment maneuvers, unconventional ventilation modes, and extracorporeal membrane oxygenation (ECMO), are employed in refractory hypoxemic cases [16]. Refractory hypoxemia requiring salvage therapies has been on the rise since the COVID-19 pandemic [17].

The focus of our article is to review salient inhaled pulmonary vasodilators utilized in CARDS and review the updated literature since the onset of the COVID-19 pandemic.

### 2.5. Principles of Inhaled Pulmonary Vasodilators

ARDS causes diffuse alveolar damage, which induces pulmonary edema and atelectasis of the alveoli due to the loss of the surfactant. It impairs oxygenation, leading to hypoxemia in the pulmonary vasculature. To maintain oxygenation, reflex pulmonary vasoconstriction occurs at the level of inflamed alveoli, thus shunting the blood from unhealthy lungs to well-ventilated alveoli of the lung [18]. The inflammatory milieu around the affected alveoli can reverse the hypoxic pulmonary vasoconstriction, shunting the blood from healthy alveoli towards damaged alveoli and further exacerbating the V/Q mismatch. Inhaled pulmonary vasodilators lead to selective pulmonary vasodilation of well-ventilated alveoli, improving oxygenation by diverting more blood to the healthy alveoli. As ARDS is a heterogenous pathology, inhaled vasodilators also reverse normal pulmonary hypoxic vasoconstriction in healthy alveoli. ARDS also increases pulmonary vascular resistance due to pulmonary vasoconstriction, atelectasis, and compression of the pulmonary vasculature due to consolidations in the lung, resulting in pulmonary hypertension and increased right ventricle (RV) afterload [19]. Inhaled pulmonary vasodilators decrease the pulmonary vascular resistance and RV afterload, hence improving the RV function.

**Figure 3:**
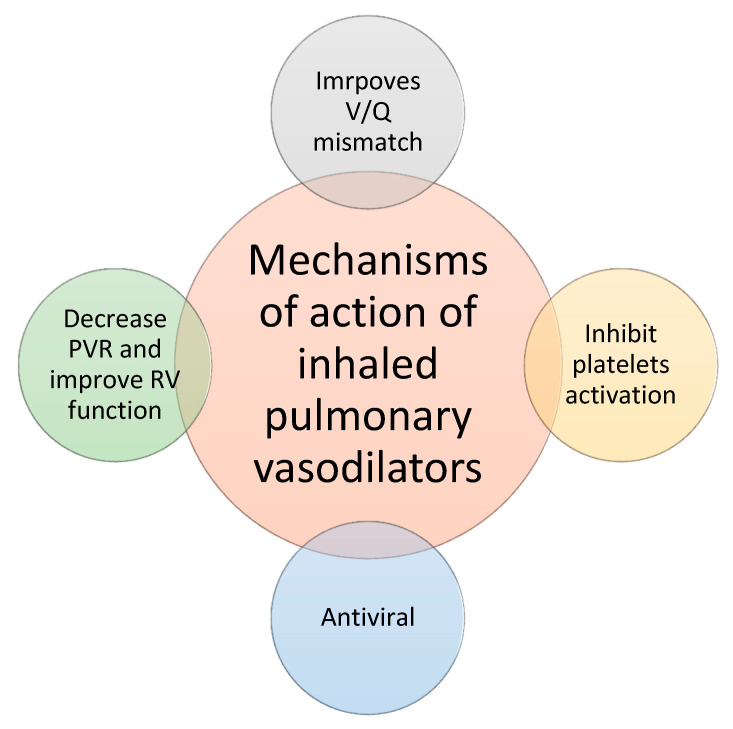
Various proposed mechanisms of action of inhaled pulmonary vasodilators. V/Q: ventilation/perfusion, PVR: pulmonary vascular resistance, and RV: right ventricle.

Especially in those with CARDS, ARDS can result in platelet hyperactivity, which may explain the high incidence of platelet-rich microvascular thrombi in pulmonary and systemic circulation [20]. Inhaled prostaglandins specifically inhibit platelet activation, hence decreasing the risk of microthrombi, which might offer adjunctive benefits in CARDS [21]. Figure 3 shows the proposed beneficial mechanisms of inhaled pulmonary vasodilators.

The most common cause of death in ARDS is multiorgan failure, but around 10–15% of ARDS-related deaths are attributed to refractory hypoxemia [16]. The use of inhaled pulmonary vasodilators is known to improve oxygenation, but so far, it has not demonstrated a mortality benefit in ARDS [16,22].

Below, n depth, we discuss two main categories of inhaled pulmonary vasodilators: inhaled nitric oxide (NO) and inhaled prostaglandins.

## 3. Inhaled Nitric Oxide

### 3.1. Introduction

Nitric oxide (NO) was first reported in 1987 as an important endothelium-derived relaxing factor [23]. In mammalian cells, it is produced by nitric oxide synthase (NOS) via the oxidation of _L_-arginine into NO and _L_-citrulline. NOS has three different isoforms in mammalian cells: endothelial NOS (eNOS), neuronal NOS (nNOS), and inducible NOS (iNOS). nNOS and eNOS have short-lived actions, but iNOS produces large quantities of NO in the setting of an infection and has a prolonged duration of action [24]. Inhaled NO is a colorless, odorless gas that is delivered to the patient in a solution form. Due to its volatile chemical nature, it has a very short duration of action.

### 3.2. Mechanism of Action

The pulmonary vasodilatory effect of inhaled NO was first demonstrated in lamb models in 1991 [25]. It causes a dose-dependent decrease in pulmonary vascular resistance (PVR) that can be immediately reversed on discontinuation [26]. NO stimulates soluble guanylate cyclase, which produces excess cyclic guanine monophosphate (cGMP). cGMP activates protein kinase G, which decreases the intracellular calcium through various downstream pathways, resulting in vascular and nonvascular smooth muscle relaxation [26]. It also causes bronchodilation and is known to have an in vitro antiviral effect due to its free radical nature [24,27]. In infectious states, proinflammatory cytokines induce NO production, having a direct antiviral effect due to their ability to inhibit viral replication and produce several reactive oxygen species, such as peroxynitrite, dinitrogen trioxide, and nitrogen oxide. It also creates oxidative stress, which can result in tissue damage. Hence, concentrated nitric oxide can offer antiviral effects but does not increase the risk of tissue injury (its optimal dose is unknown) [24]. The most prominent effect of nitric oxide in ARDS is selective pulmonary vasodilation in adequately ventilated areas, which decreases intrapulmonary shunting and, hence, improves oxygenation.

### 3.3. Metabolism of NO

Nitric oxide quickly diffuses across the alveolocapillary membrane and reacts with oxyhemoglobin to form methemoglobin and nitrate. Within 48 h of the inhalation of NO, 70% is excreted as nitrate in urine [22]. The methemoglobin levels should be checked before starting iNO and monitored with the continued use of NO. Methemoglobinemia is noted to be more pronounced at dosages greater than 50 parts per million (ppm).

### 3.4. Adverse Effects

In general, the inhalation of nitric oxide is considered safe. Due to its short half-life, the adverse effects are short-lived. Below are the most common adverse effects.

#### 3.4.1. Hypotension

Although inhaled nitric oxide is a pulmonary vasodilator, it is also reported to cause systemic vasodilation via the binding of nitric oxide to circulating albumin or hemoglobin. Albumin-bound nitric oxide is biologically active and produces systemic venous, arterial, and coronary arterial vasodilation [28]. Nitric oxide can also bind to vacant heme sites in hemoglobin and nitrosylate thiols without being activated. Albumin and hemoglobin deliver active nitric oxide into the systemic circulation; consequently, systemic vasodilation can result in hypotension.

#### 3.4.2. Methemoglobinemia

Nitric oxide has an affinity for hemoglobin about 1500 times greater than carbon monoxide [28]. Inhaled nitric oxide combines with hemoglobin to form nitrosyl hemoglobin, which rapidly oxidizes to methemoglobin. Methemoglobin does not readily release oxygen, which eventually can lead to tissue hypoxia. The management of these patients includes methylene blue in those who have MetHb levels greater than 30% or >20% in symptomatic patients [29].

#### 3.4.3. Platelet Inhibition and Prolonged Bleeding Time

Several animal and human studies shave shown that inhaled nitric oxide increases the bleeding time and inhibits platelet aggregation [30]. Since nitric oxide is delivered to the systemic circulation, this likely occurs via the nitric oxide activation of soluble guanylate cyclase, which catalyzes the formation of cGMP from GTP. An increased cGMP concentration activates cGMP-dependent protein kinase type I (cGKI). cGKI-dependent substrate phosphorylation limits platelet activation and thrombus formation by inhibiting intracellular calcium release, integrin activation, cytoskeletal remodeling, and granule secretion, thereby causing prolonged bleeding time [30].

#### 3.4.4. Pulmonary Edema in Heart Failure

In patients with heart failure, the left ventricle (LV) is typically noncompliant. Inhaled nitric oxide has been reported to increase LV end-diastolic pressures due to the augmentation of filling into a relatively noncompliant LV. Inhaled nitric oxide causes pulmonary vasodilation and may overwhelm an already failing left ventricle, causing pulmonary edema and therefore hypoxia [22].

#### 3.4.5. Rebound Hypoxia and Pulmonary Hypertension on Withdrawal

The administration of inhaled nitric oxide can result in decreased endogenous nitric oxide synthesis. The consequent upregulation of vasoconstrictor molecules such as endothelin occurs to balance the vasodilator effect of NO. This can not only decrease nitric oxide’s effectiveness over several days, but a sudden cessation of inhaled nitric oxide can also cause severe rebound pulmonary hypertension, an increase in intrapulmonary right-to-left shunting, and decreased PaO_2_ [22,31]. A case report described a refractory post-op pulmonary hypertension that occurred following NO discontinuation. The patient was then treated with dipyridamole, a c-GMP-specific phosphodiesterase (PDE) 5 inhibitor, which led to the successful discontinuation of NO [32]. At this time, this is primarily anecdotal, and further research is needed to assess the utility and safety of dipyridamole or other agents in similar cases.

#### 3.4.6. Renal Failure

The mechanism of renal failure in iNO is somewhat unclear. Some animal and human studies have proposed that nitric oxide inhalation increases the serum levels of cGMP, nitrite, and nitrates, causing protein nitrosation and raising the oxidative load that plays a role in the development of acute kidney injury (AKI) [33]. Additionally, ARDS patients receiving iNO usually receive high concentrations of oxygen, facilitating the production of reactive nitrogen species that increase oxidative injury and AKI [33].

### 3.5. Weaning of Inhaled Nitric Oxide

Nitric oxide should be weaned from rather than an abrupt discontinuation to decrease the likelihood of rebound pulmonary hypertension and rebound hypoxemia. In general, it is recommended to wean from nitric oxide when the ventilator and inotropic support have significantly reduced or after 96 h of therapy [34]. 

The typical dose for iNO is 20 parts per million (ppm). Before discontinuation, the dose should be reduced to 1 ppm for 30 min to an hour. Weaning should be attempted every 12 h [34].

### 3.6. How to Administer Inhaled Nitric Oxide

Inhaled nitric oxide is administered via an endotracheal tube, nasal cannula, high-flow nasal cannula, or nasal prongs. Via an endotracheal tube, it can be administered downstream the ventilator into the inspiratory limb of the ventilatory circuit or mixed with oxygen and nitrogen into the upstream part of the system. Administration downstream is reported to be cheaper and is more commonly used in Southern Europe, whereas upstream administration is more common in the United States and Northern Europe [35]. Opti-NO is a system designed for the sequential administration of NO into the ventilator downstream. iNOmax is another delivery system for nitric oxide gas. Delivery via nasal prongs can have significant gas dilution and require higher concentrations of nitric oxide (up to 50 ppm) [31].

## 4. Inhaled NO Use in the COVID Era

The spectrum of COVID-19 infection ranges from asymptomatic illness to severe ARDS associated with significant mortality [36]. Out of the 20–30% of hospitalized patients, a severe disease requiring ICU care and advanced ventilatory support occurs in about 4.9–11% of hospitalized patients, and severe ARDS develops in about 3.4% [37]. Mortality and morbidity in CARDS depend upon multiple factors, including age; comorbidities such as diabetes, malignancy, and an immunocompromised state [38]; different variants of the disease (i.e., Omicron); and access to early medical intervention [39,40,41].

Once ARDS settles into COVID-19 patients with hypoxemia, invasive mechanical ventilation (IMV) becomes inevitable [42,43]. In cases with refractory hypoxia, rescue therapies and recruitment maneuvers have been utilized as either alternatives or adjuncts to IMV to improve oxygenation in COVID-19-diseased lungs [44].

Traditionally, pulmonary vasodilators, i.e., inhaled nitric oxide (iNO), have been used in ARDS as salvage therapy with variable outcomes [45]. In 2016, a large meta-analysis of 14 randomized control trials, including children and adults with severe ARDS, demonstrated no statistically significant benefit in the survival rate, ventilator-free days, or mortality benefit in the iNO-treated group vs. the controls (placebo or no intervention). However, there was significant improvement in the PaO2/FiO2 ratio up to 24 h and improvement in the oxygenation index [46]. However, most published studies pose a relatively modest risk of bias, as they suffer from a certain number of methodological problems that were insufficiently defined and varied greatly from one study to another. These include a lack of blinding; explicit protocol for ventilator management; inadequate statistical power; heterogenous patient population; ARDS etiology; modalities of administration (concentration, duration, evaluation of the response, and weaning); and heterogenous and ill-defined protocol for monitoring [46,47,48].

Given a favorable benefits/risk ratio, iNO continues to be used in patients with severe ARDS. The physiological benefits (i.e., reduction of ventilation-perfusion mismatch, improved oxygenation, and right ventricular performance) justify its use, especially when used with adjunct therapies such as high-frequency oscillatory ventilation (HFOV) or prone positioning when the optimal mechanical ventilation does not correct hypoxemia [49,50].

### 4.1. Role of iNO in Moderate to Severe CARDS

The case fatality of COVID-19 is averaging about 2–3% worldwide [51]. Of those, 30.8% report CARDS as a leading cause of death [52]. The Surviving Sepsis campaign and the European Respiratory Society recommend a trial of iNO as a short-term rescue therapy with a quick taper when there is no improvement in CARDS [44,53].

Chen and his colleagues demonstrated the first use of nitric oxide for SARS Coronavirus infection during the SARS epidemic of 2002 to 2003. Their findings suggested improvement in oxygenation and a lasting effect on the disease after the termination of NO [54]. A study followed this in Leuven, Belgium, in 2004, showing an in vitro inhibition of SARS Coronavirus cytopathic effects by 50% with one of the NO donor compounds, S-nitroso-N-acetyl penicillamine [55]. In 2005 and 2009, two studies published by the Swedish Institute of Infectious Disease exhibited a dose-dependent inhibitory effect of NO on the replication cycle of SARS Coronavirus, especially in the early stages of infection [56,57].

A prospective study at Picardie University Hospital in Amiens, France, was performed in March 2020, early in the COVID-19 pandemic. Eighty patients with severe COVID pneumonia (defined by a PF ratio < 150) received iNO at 10 ppm, with a goal response of a 20% increase in the PF ratio over 30 min. The results indicated that 65% of the patients responded to the therapy. Notably, a lower-baseline PF ratio was noted in responders vs. non-responders (i.e., a mean of 70 (CI 63–100) vs. 134 (CI 83–173)) [58]. Similarly, a small retrospective analysis published in August 2020 evaluated seven patients with CARDS who received 20 ppm iNO for 20–30 min and indicated improved oxygenation [59].

In May 2020, Tufts Medical Center carried out a pilot randomized controlled trial investigation of inhaled NO to prevent progression to a more advanced disease in 42 hospitalized patients with COVID-19. The experimental group received iNO via an iNO pulse device at a dose of 125 mcg/kg IBW/h. The primary outcomes included the prevention of the progression of the disease severity within 28 days of hospitalization. The study was terminated early, because the small data set invalidated the outcomes. However, the results reported low severity scoring in patients treated with iNO vs. the controls (Table 2, NCT04397692) [60].

In October 2020, a single-center retrospective case–control study was conducted at the University College of London to determine the effects of inhaled NO in patients with CARDS vs. ARDS. The results showed improvement in their PF ratio in only 40% of CARDS patients vs. 77% of ARDS due to other causes. The poor response to iNO-induced pulmonary vasodilation in CARDS was postulated to be secondary to COVID-induced lung injury vasodilation. However, the study was limited, given its small sample size and the use of iNO at variable stages of ARDS [61]. In December 2020, a clinical trial of iNO in pregnant patients with severe COVID-19 at Massachusetts General Hospital reported that six pregnant patients admitted with critical COVID-19 received high-dose iNO (160–299 ppm) for a total of 39 treatments. The results indicated improvement in cardiopulmonary function and systemic oxygenation with a reduction in tachypnea. Three patients delivered a total of four neonates during hospitalization. They concluded that high-dose iNO is safe, well-tolerated, and may benefit pregnant patients with hypoxemic respiratory failure [62,63].

Multiple randomized clinical trials whose results have yet to be published are currently being held in different phases of completion in different regions of the world. Our limited data regarding the use of inhaled NO in CARDS suggests improvement in oxygenation but no survival benefit. The details of the trials are given in Table 2 [60].

### 4.2. Role of iNO in Mild COVID-19 Infection

Given the antiviral properties of nitric oxide [55,56,57], several studies have been performed to treat and decrease the viral load in patients with mild COVID infection to prevent disease progression and spread. Baek et al. [64] investigated the duration of viral shedding in COVID-19-infected patients and demonstrated that viral clearance takes no longer than 15 days post the onset of symptoms. This was followed by another study published in 2021 indicating a median viral clearance of 13 days that can lag for as long as 20 days [65]. Therefore, multiple clinical trials have utilized the idea of reducing viral shedding, increasing viral clearance, and preventing spread.

In August 2021, Winchester and colleagues published a study including 80 adults (18–70 years) with mild COVID infection (confirmed by RT-PCR) who were treated with nitric oxide nasal spray (NONS) four days after the onset of symptoms. A rapid reduction (95%) in the SARS-CoV-2 viral load was noted within 24 h, with a 99% reduction within 72 h of NONS treatment [66]. In a small study conducted at the Boston University School of Medicine, 39 spontaneously breathing COVID-19 patients were treated with 30 ppm iNO with a mean duration treatment of 2.1 days. Over half the patients (53.9%) did not require invasive mechanical ventilation and showed improvement in spO_2_/FiO_2_ after treatment with iNO [67].

Interestingly, tankless iNO delivery systems have demonstrated improvement in dyspnea and a progressive increase in home-administered six-minute walk tests, although a single case demonstrated the potential use of a portable NO inhalation system to treat patients with mild COVID infection at home [68,69].

Given the utility of antiviral inhaled NO, there are multiple ongoing trials in Arkansas, Canada, Africa, and multiple other centers using different nitric oxide preparations and routes to prevent the progression of mild COVID infection (Table 2) [60].

### 4.3. Preventive Role of NO and Limitations of Its Use

As the COVID pandemic infected millions of people worldwide, effective preventative measures, including handwashing, mask-wearing, and physical distancing, were associated with reductions in incidence [70]. Since then, NO has been acknowledged to have a documented role in prevention and transmission related to early viral clearance and host recovery. Multiple clinical trials have speculated on using exogenous nitrous oxide in disease prevention, especially in high-risk populations such as healthcare workers [60].

Currently, there are four ongoing trials in different phases, including two trials at Mass General Hospital and the Imperial College of London. The primary endpoint of each trial is to determine the COVID positivity with RT-PCR in patients treated with a different formulation of nitric oxide (Table 2) [60].

The data regarding the use of inhaled NO in ARDS and CARDS vary in efficacy, safety, and cost-effectiveness. A recent meta-analysis assessing the currently available literature on the use of inhaled pulmonary vasodilators in CARDS does suggest improvement in oxygenation. Still, there is no difference in mortality, lung compliance, need for endotracheal intubation, or hospital length of stay [71]. This is crucial, especially in patients with severe ARDS, for whom the goal of oxygenation takes priority over the antiviral properties of iNO.

With the ongoing clinical trials, the therapeutic effects of iNO are still uncertain. Dosing regimens with safety and efficacy have not yet been defined conclusively, although the results of continued trials have yet to be published. Nevertheless, given the theoretical benefits and minimal side effects, NO continues to be a salvage therapy for many patients with severe ARDS.

## 5. Inhaled Prostaglandins

### 5.1. Introduction

Inhaled prostaglandins such as epoprostenol and iloprost have been used increasingly in various pulmonary diseases, including ARDS and pulmonary arterial hypertension, to reduce the resistance of pulmonary vasculature and improve ventilation–perfusion mismatching, leading to improved oxygenation. 

Prostaglandin I2, otherwise known as prostacyclin or epoprostenol, interacts with the prostaglandin I receptors via a cyclic adenosine monophosphate pathway, leading to the relaxation of smooth muscle, which causes vasodilation. This particularly inhaled prostaglandin also has antithrombotic and anti-inflammatory effects. It has been used in ARDS and pulmonary hypertension with evidence of right heart failure [72]. 

### 5.2. Metabolism of Inhaled Prostaglandins

Cost-effective alternatives to nitric oxide include aerosolized systemic prostacyclins, such as epoprostenol, and synthetic analogs such as iloprost and treprostinil. Epoprostenol was the first widely available inhaled prostacyclin, but its short half-life has made it difficult to deliver effectively. Synthetic analogs were created in an attempt to stabilize the molecule to enable more reliable dosing. Prostacyclin analogs such as epoprostenol are metabolized via both enzymatic formation and spontaneous degradation into active metabolites, which are then consumed and then extensively metabolized via urinary excretion. 

Synthetic prostacyclin analogs, such as iloprost and treprostinil, have previously been used as a treatment for pulmonary arterial hypertension. They inhibit myosin light-chain kinases, which cause smooth muscle relaxation with high bioavailability. Iloprost is primarily metabolized hepatically via beta-oxidation of the carboxyl side chain [73] and has a half-life of 30–60 min [74]. Inhaled treprostinil has a half-life of up to 4 h and undergoes oxidative hepatic metabolization [75,76].

### 5.3. Adverse Events of Inhaled Prostaglandins

Inhaled prostaglandins and their analogs have several on-target and off-target adverse drug reactions. As they are potent vasodilators, a significant concern is a systemic hypotension. When given systemically, epoprostenol causes hypotension, meaning its effects are not specific to pulmonary tissue. To mitigate systemic hypotension when treating ARDS, prostaglandins are given as inhaled formulations. While many prospective studies show a low frequency of systemic hypotension, the cohort analysis showed up to 17.4% of patients can experience systemic hypotension [21]. While this data are limited and true incidence is unclear, there have been measured prostaglandin metabolites (6-keto PGF1α) found in systemic circulation after inhaled prostaglandin delivery [77]. There is heightened concern for systemic hypotension if ARDS patients also have LV dysfunction, which could quickly lead to vascular congestion and pulmonary edema. Pulmonary edema is often viewed as a relative contraindication to inhaled prostaglandins. Finally, there is a concern for tolerance to inhaled iloprost after prolonged exposure [78].

Several other adverse effects have been reported in clinical settings, such as cough, dizziness, headache, and flushing [79]. The most feared clinical complications of inhaled prostaglandins and their synthetic derivates come from rebound pulmonary hypertension from rapid dose de-escalation.

### 5.4. Weaning of Inhaled Prostaglandins

It is important to have a weaning protocol for inhaled prostaglandins. There is a concern for severe rebound pulmonary hypertension if the medication is stopped or the dose is reduced too quickly [80]. One protocol from Washington University cites dose-reducing epoprostenol by 50% every two to four hours; there was no reported rebound hypertension in their 126-person cohort [81].

### 5.5. Equipment for Inhaled Prostaglandins Use

Epoprostenol has a very short half-life (two to three minutes) and so requires continuous nebulization [82]. It is initially delivered as salt and reconstituted with a glycine buffer dilutant. Doses can either be fixed, with an upper limit of 160,000 ng/h, or figured by weight, with an upper limit of 50 ng/kg/min [81,83]. Regardless, the solution should run at six to eight ml/h into the nebulizer to maintain a 15-mL priming capacity. An O_2_ blender is used at two to three L/min, and the nebulizer should be connected to a heated wire circuit.

For intubated patients with endotracheal tubes, there are several potential places to introduce the aerosol: at the humidifier inlet, humidifier outlet, or within the inspiratory limb of the circuit between the endotracheal tube and the Y-piece. Limited studies looking into nebulizer placement in the circuit have failed to show any difference in drug delivery or impact on the patient [82]. A filter is placed in the exhalation tube to prevent the aerosol from entering the exhalation circuitry. For awake, non-intubated patients, a face mask with a well-sealed air cushion is used. The aerosol is administered via a T-connector in a single-tube circuit with a filter for exhaled air to prevent aerosol from dispersing into the atmosphere. The glycine buffer is known to clog filters, so they must be changed every two to four hours, as continuous medication delivery is critical. It is important to note that drug delivery can be impacted by ventilator settings (respiratory rate, PEEP, and tidal volume); the type and size of tubing used; and the nebulizer and ventilator types [83].

Synthetic prostacyclin analogs are generally delivered in bolus dosing instead of continuously, which is feasible due to their longer half-lives. Iloprost is delivered via a nebulizer, and the dose frequency is based on the nebulizer technology utilized. Iloprost was initially delivered via jet nebulizers to deliver the medication continuously. Then, ultrasound nebulizers and aerosol delivery systems were used to deliver the medication during inhalation selectively. This shortens the overall treatment time and maximizes the proportion of the drug that is delivered. I-net is a vibrating mesh nebulizer coupled with an adaptive aerosol delivery system that provides medication with each inhale. These systems have mainly been replaced by a vibrating mesh nebulizer that further reduces drug waste [84]. Even vibrating mesh nebulizers delivering iloprost require six to nine doses/per day, and each dose can take up to 12 min [85]. While iloprost is generally delivered via handheld nebulizers, ongoing trials are investigating using it with intubated patients on a ventilator [86].

Treprostinil has the most diverse medication forms of prostacyclin analogs. It can be delivered orally, subcutaneously, intravenously, or inhaled. Inhaled treprostinil can be administered via an ultrasonic nebulizer, with three breaths delivered four to nine times daily with 6 ug of medication delivered each breath [87]. The only FDA-approved delivery system is the ultrasonic nebulizer Tyvaso inhalation system. It is not used widely for intubated patients on a ventilator, although it is being studied in simulations and in vitro mechanical ventilation models [88].

Alternative strategies of rapid bolus inhalation, where each dose is administered in one to two breaths as an inhaler or MDI, are being investigated [79].

### 5.6. Inhaled Prostaglandins Use in the COVID Era

Inhaled prostaglandins have been historically used for severe ARDS, in addition to other survival-improving treatments such as low tidal volume ventilation [45,89], prone positioning [90], and a neuromuscular blockade [91]. Similar to inhaled NO, inhaled prostaglandin has been shown to have pulmonary vasodilatory, broad anti-inflammatory, and antiplatelet effects [92]. It is therefore conceptualized that inhaled prostaglandin (i.e., epoprostenol, iloprost, and treprostinil) can improve arterial oxygenation by improving ventilation–perfusion mismatches similar to iNO but have a better cost vs. benefits profile [93].

In 2015, a systemic review and meta-analysis including 25 studies with two randomized controlled trials (RCTs) over 21 years (1993–2014) demonstrated improved oxygenation, pulmonary artery pressures, and some associated side effects [21]. A Cochrane database review of two RCTs in 2017 that included 14 children failed to identify a clear benefit in using aerosolized prostacyclin to improve oxygenation in patients with severe ARDS due to a lack of data [94]. Although a clinical benefit has not yet been conclusively shown, inhaled prostaglandins have been associated with pharmacological properties that affect the underlying pathology of ARDS [95,96] and improve hypoxia with decreased oxygen supplementation, reducing free radicular damage to lung parenchyma [97].

In October 2020, an RCT treated 38 critically ill COVID-19 and refractory hypoxemia patients with either epoprostenol or inhaled NO for one hour. On average, the results did not show any improvement in the oxygen metrics. However, the responders significantly improved their PF ratios, and patients receiving both inhaled epoprostenol and iNO had minimal side effects [98]. In a recent retrospective observational study on 80 mechanically ventilated patients at two tertiary care centers, nearly 50% of the patients were noted to have a significant clinical improvement in their PF ratios with modest overall benefits when used conjointly with prone positioning [99].

Another retrospective study investigating the combined benefits of prone ventilation and inhaled epoprostenol reported a more than 20% improvement in combination therapy and lower mortality rates (52% vs. 81% *p* = 0.02) between responders and non-responders [100].

Given the emergent need for better therapeutics for the treatment of COVID-19 lung disease, different prospective and randomized trials are being carried out, including using VentaProst on mechanically ventilated patients at Ohio State University Columbus, a COMBAT-COVID trial in Denmark, and a study of ILOCOVID at Hamad Medical Corporation in Qatar that uses prostaglandins to determine their efficacy in terms of better outcomes associated with CARDS (Table 3) [101].

## 6. Conclusions

Based on the currently available evidence, inhaled pulmonary vasodilators remain a salvage therapy in refractory hypoxia in classic ARDS and CARDS. It has been shown to improve oxygenation but has no survival benefit. Due to its antiviral effects, inhaled nitric oxide may offer a preventive role in viral infections. Further large randomized controlled trials are still needed to elucidate the role of inhaled pulmonary vasodilators in the management of various severities of ARDS.

## Figures and Tables

**Table 1:** Key difference between CARDS and typical ARDS.

	COVID-ARDS	Typical ARDS
**Onset**	Within 8 to 12 days	Within 7 days
**Immune cells**	Peripheral ground glass opacities in early disease	Patchy diffuse bilateral dependent consolidations
**Immune cells**	Monocytes, lymphocytes, and neutrophils	Predominantly neutrophilic infiltrate
**Immuno-thrombosis**	More prevalent likely due to diffuse endothelial injury from a hyperinflammatory state	Less prevalent as compared to CARDs

**Table 2:** Nitric oxide (NO) therapy under various stages of clinical trials for COVID-19 treatment.

Identifier	Brief Title	Severity	Study Design	Drug	Dose	Duration	Subjects	Follow-Ups	Study Phase	Status
NCT04388683	Inhaled NO for preventing progression in COVID-19	Moderate-severe	RCT	NO gas	125 mcg/kg (20 ppm)	24 h	42	28	Phase 2	Completed
NCT04383002	High dose inhaled NO for COVID-19 (ICU patients)	Moderate-severe	RCT	NO gas	160 ppm, once	6 h	7	7	Phase 1	Recruiting
NCT04305457	NO gas inhalation therapy for mild/moderate COVID-19	Moderate-severe	RCT	NO gas	140–180 ppm, twice	20–30 min	70	28	Phase 2	Recruiting
NCT04456088	Inhaled NO for treatment of COVID-19 caused by SARSCoV2 (Canada trial)	Moderate-severe	RCT	NO gas	80 ppm, four times	40 min	50	14	Phase 2/1	Not yet recruiting
NCT04337918	NO-releasing solutions to prevent and treat mild/moderate COVID-19 infection	Mild-moderate	RCT	NO gas	Five times	14 days	200	14	Phase 2	Recruiting
NCT04421508	A study to assess pulsed inhaled NO vs. placebo in subjects with mild or moderate COVID-19	Mild-moderate	RCT	NO gas	125 mcg/kg (20 ppm)	24 h	500	28	Phase 2	Recruiting
NCT04476992	NO therapy for COVID-19 patients with oxygen supplementation	Mild-moderate	RCT	NO gas	200 ppm, twice	30 min	20	14	Phase 2/1	Not yet recruiting
NCT04397692	Inhaled NO for the treatment of COVID-19 caused by SARSCov-2 (US trial)	Mild-moderate	RCT	NO gas	80 ppm, four times	40 min	20	14	Phase 3	Recruiting
NCT04306393	NO gas inhalation in SARS in COVID-19	Mild	RCT	NO gas	80 40	48 h	200	28	Phase 2	Recruiting
NCT04312243	NO prevention of COVID-19 for healthcare providers	Close contact	RCT	NO gas	160 ppm, twice	15 min	470	14	Phase 2	Recruiting
NCT04601077	The evaluation of NO generating lozenges on the outcome of newly diagnosed COVID-19 African Americans patients	Mild	RCT	NO lozenges	30 mg, twice	30 days	100	30	Phase 1	Not yet recruiting
NCT04388683	Inhaled NO for preventing progression in COVID-19	Moderate-severe	RCT	NO gas	125mcg/kg (20 ppm)	24 h	42	28	Phase 2	completed
NCT04383002	High dose Inhaled NO for COVID-19 (ICU patients)	Moderate-severe	RCT	NO gas	160 ppm, once	6 h	7	7	Phase 1	Recruiting
NCT04305457	NO gas inhalation therapy for mild/moderate COVID-19	Moderate-severe	RCT	NO gas	140–180 ppm, twice	20–30 min	70	28	Phase 2	Recruiting
NCT04456088	Inhaled NO for treatment of COVID-19 caused by SARSCoV2 (Canada trial)	Moderate-severe	RCT	NO gas	80 ppm, four times	40 min	50	14	Phase 2/1	Not yet Recruiting
NCT04337918	NO-releasing solutions to prevent and treat mild/moderate COVID-19 infection	Mild-moderate	RCT	NO gas	five times	14 days	200	14	Phase 2	Recruiting
NCT04421508	A study to assess pulsed inhaled NO vs. Placebo in subjects with mild or moderate COVID-19	Mild-moderate	RCT	NO gas	125 mcg/kg (20 ppm)	24 h	500	28	Phase 2	Recruiting
NCT04476992	NO therapy for COVID-19 patients with oxygen supplementation	Mild-moderate	RCT	NO gas	200 ppm, twice	30 min	20	14	Phase 2/1	Not yet Recruiting
NCT04397692	Inhaled NO for the treatment of COVID-19 caused by SARSCov-2 (US trial)	Mild-moderate	RCT	NO gas	80 ppm, four times	40 min	20	14	Phase 3	Recruiting
NCT04306393	NO gas inhalation in SARS in COVID-19	mild	RCT	NO gas	80 40	48 h	200	28	Phase 2	Recruiting
NCT04312243	NO prevention of COVID-19 for healthcare providers	Close contact	RCT	NO gas	160 ppm, twice	15 min	470	14	Phase 2	Recruiting
NCT04601077	The evaluation of NO generating lozenges on the outcome of newly diagnosed COVID-19 patients in African Americans	mild	RCT	NO lozenges	30 mg, twice	30 days	100	30	Phase 1	Not yet Recruiting

**Table 3:** Inhaled prostaglandin therapy under various stages of clinical trials for COVID-19 treatment.

Identifier	Brief Title	Severity	Study Design	Drug	Dose	Duration	Subjects	Follow Ups	Study Phase	Status
NCT04388683	VentaProst in subjects with COVID-19 requiring ventilation	Moderate-severe	RCT; Double-blind	Inhaled epoprostenol	N/A	10 days	11	10 days	Complete	Results pending
NCT04383002	Infusion of iloprost vs. placebo for 72 h in COVID-19 respiratory failure	Moderate-severe	RCT	Iloprost	1 ng/kg	24 h	80	28 days	Complete	Results pending
NCT04338828	Inhaled iloprost for suspected COVID-19 respiratory failure	Moderate-severe	RCT	Iloprost	20 mcg	Every 8 h	40	5 days	Complete	Results pending

## Data Availability

Not applicable.
